# Organ donation and brain preservation after assisted dying: case report and considerations for a potential pathway

**DOI:** 10.1186/s12910-026-01586-1

**Published:** 2026-09-01

**Authors:** Alexander German, Alexander Grotemeyer, Katharina Koller, Markus Eckstein, Hannes Wolff, Stefan Rampp, Patrick Süß, Vincent C. Müller, Dieter Birnbacher, Jürgen Winkler, Martin Regensburger

**Affiliations:** 1 Department of Molecular Neurology, University Hospital Erlangen, Friedrich- Alexander-Universität Erlangen-Nürnberg, Erlangen, Germany; 2 Institute of Neuroradiology, University Hospital Erlangen, Friedrich-Alexander- Universität Erlangen-Nürnberg, Erlangen, Germany; 3 Klinikum Nürnberg, Neurologie, Paracelsus Medizinische Privatuniversität, Nürnberg, Germany; 4 Department of Psychosomatic Medicine and Psychotherapy, University Hospital of Erlangen, Friedrich-Alexander-University Erlangen-Nürnberg, Erlangen, Germany; 5 Department of Pathology, University Hospital Erlangen, Friedrich-Alexander- Universität Erlangen-Nürnberg, Erlangen, Germany; 6 Federal Constitutional Court, Karlsruhe, Germany; 7 Department of Neurosurgery, University Hospital Erlangen, Friedrich-Alexander- Universität Erlangen-Nürnberg, Erlangen, Germany; 8 Centre for Philosophy and AI Research, Friedrich-Alexander-Universität Erlangen-Nürnberg, Erlangen, Germany; 9 Institute for Philosophy, Heinrich-Heine-Universität Düsseldorf, Düsseldorf, Germany

**Keywords:** Medical assistance in dying, Physician-assisted suicide, Organ donation, Donation after circulatory death, Brain banking, Structural Brain Preservation, Cryonics, Cryothanasia, Amyotrophic lateral sclerosis.

## Abstract

Protecting life and restoring health have been at the core of the conception of medical care since antiquity, for health professionals and the public alike. Assisted dying represents a practice that appears to challenge this traditional conception. In jurisdictions where assisted dying is condoned, the question remains as to whether the practice should be medicalized or deferred to non-medical actors. Irrespective of the divisive controversy surrounding assisted dying and its medicalization per se, the practice gives rise to high-stakes ethical, legal and organizational challenges when intersecting with organ transplantation, tissue banking, and Structural Brain Preservation or cryonics. In this narrative account, we describe the contextual framework and outline our experience and operational scope for addressing them at a university hospital in Germany. Based on this description we propose a structured pathway for increasing donation and preservation options for terminal patients while retaining ethical integrity and professional accountability.

## Introduction

Vesalian anatomy since the 16th century and Virchowian pathology since the 19th have established access to the deceased human body as a cornerstone for medical research and education [1, 2]. As investigational methods advance alongside the capacity for ex-vivo maintenance of biological cells, tissues and organs, the requirements for scientific biomaterials now approach the standards established for organ transplantation during the 20th century [3]. In principle, the ongoing rise in prevalence of assisted dying across several Western nations in the 21st century facilitates access to viable tissues and organs from consenting donors based on their autonomous choices [4]. In particular, assisted dying may not only represent an ethical access to transplantable organs, but also to the viable human brain in its totality, e.g., for connectomic studies and preservation practices [5–8].

Here, we analyze motives to establish immediate medical access to the deceased human body from the three ethically distinct but procedurally interconnected domains (i) organ donation, (ii) research and (iii) Structural Brain Preservation (SBP) or cryonics practices. As a starting point, we report the clinical case of a patient with neurodegeneration who decided to undergo medically assisted suicide, and in whom the autonomous wish for all three practices (i)-(iii) coincided. From a technical standpoint, except for assisted dying, none of these wishes could be realized to a satisfactory degree. Based on the discussion of this paradigmatic case, we will subsequently propose a donation-preservation pathway to better account for such wishes.

## Case presentation

### Synopsis

A woman in her early 50s was diagnosed with amyotrophic lateral sclerosis (ALS). As her physical condition deteriorated, ultimately leaving her unable to speak and dependent on care, she independently arranged to end her life through assisted suicide. She asked the clinical team to combine her assisted death with organ donation for transplantation, research and SBP. The clinical team could only accommodate corneal donation and SBP following an extended post-mortem interval.

### Clinical narrative

A woman in her early 50s, with no prior medical history, presented to our outpatient clinic with dysarthria and nasal speech. The symptoms had gradually occurred and worsened over time, when she presented five months after symptom onset. A neurological examination was unrevealing except for palatal paresis causing velopharyngeal insufficiency. Magnetic resonance imaging of the brain, cerebrospinal fluid (CSF) analysis, evoked potentials and nerve conduction studies were normal. A definite diagnosis could not be established.

17 months after symptom onset, the clinical syndrome evolved into tongue fasciculations and generalized amyotrophy, including symmetric weakness in shoulder abduction, elbow flexion, and finger extension (Medical Research Council grade 4/5) and diffuse hyperreflexia with spread. Repetition of muscle ultrasonography revealed widespread fasciculations at the cranial, cervical and lumbosacral levels along with pathological spontaneous activity on electromyography of the deltoid muscle. Antineural antibodies were negative and genetic testing showed no pathogenic variants. Serum neurofilament light chain was elevated at 71.6 pg/mL (normal < 23) supporting active axonal neurodegeneration. Consequently, a diagnosis of motor neuron disease was established.

The patient was informed about the diagnosis and prognosis of sporadic, bulbar-onset ALS. A treatment with riluzole and high-calorie nutritional supplements to counteract weight loss was initiated. In alignment with standards for anticipatory guidance in progressive neurodegenerative disease, the patient received counseling for advance care planning [9]. This included recommendations to establish an advance directive (*Patientenverfügung*) and a health care proxy (*Vorsorgevollmacht*). Multidisciplinary psychosocial support included referral to a counseling center for neuromuscular disease. The clinical team committed to continual support and established a schedule for regular clinical follow-up. During the fifth follow-up visit, 25 months after symptom onset, the patient was counseled regarding brain donation options that included: (1) no donation; (2) conventional donation involving destructive tissue processing for research; (3) Structural Brain Preservation (SBP) via long-term non-destructive archiving of the specimen in the biobank of the university hospital. Regarding SBP, the patient was informed verbatim that *‘New technological possibilities may make it possible in the future to map the brain’s structure. The consequences of this are currently not foreseeable. It is speculated that*,* in the distant future*,* it might be possible after brain archiving to read out memories collected during a person’s lifetime or to restore brain function.*’^1^ The options were presented in a non-directive manner, with no recommendation provided by the clinical team. The patient selected SBP (option 3), and provided written informed consent.

**Fig. 1:**
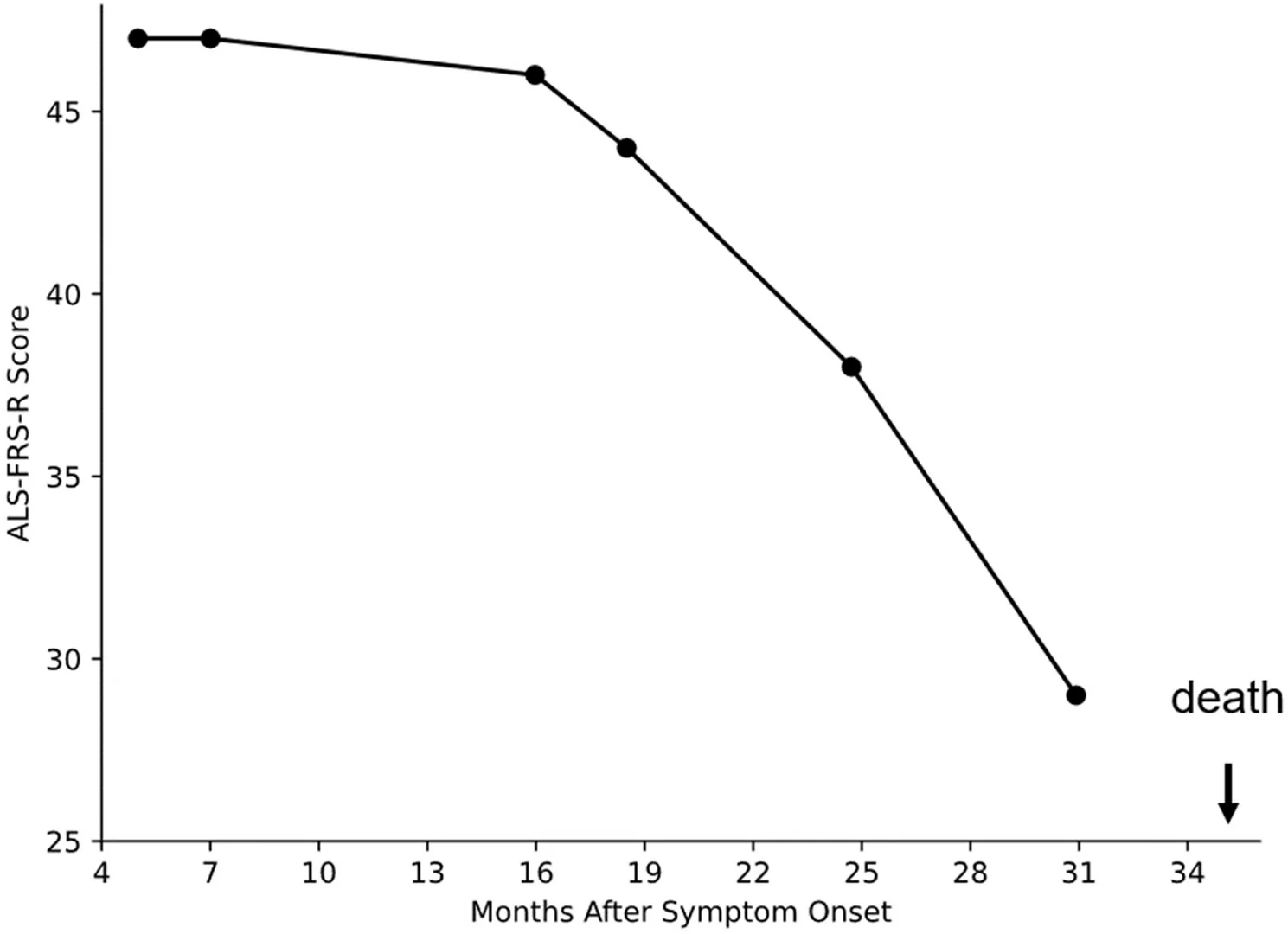
Progression of Amyotrophic Lateral Sclerosis Functional Rating Scale–Revised (ALS-FRS-R)

At the 6th visit to our outpatient clinic, at month 31 after initial symptom onset, the clinical syndrome had progressed into severe dysarthria, restricting the patient to written communication along with gait disturbance, falls, increasing disability and dependence. The corresponding clinical progression on the ALS functional rating scale (ALS-FRS-R) is visualized in Fig. 1. During the visit, on her own initiative, the patient expressed the wish to end her life. The clinical team had never previously discussed assisted dying with her. She presented to the clinical team excerpts of her correspondence with an organization offering assisted dying, seeking advice on the trustworthiness of this offer. The correspondence presented to the clinical team indicated membership fees and document processing costs of approximately €9,000.

The clinical team conducted a comprehensive consultation on the various options for advance care planning, including (i) maximal therapy with early placement of a percutaneous endoscopic gastrostomy (PEG) tube and tracheostomy ventilation once required; (ii) an observational approach without a yet further predetermination of care; (iii) adoption of a palliative treatment concept with a deliberate decision against tracheostomy; (iv) options for palliative sedation and voluntary stopping of eating and drinking (VSED); as well as (v) the general permissibility of assisted suicide in Germany. For in-depth counseling as well as an offer to establish a sustainable therapeutic team relationship, the patient was referred to a palliative care specialist.

At month 35, the patient communicated her plans for assisted dying in the following week, which she had prepared with the respective organization. On her own initiative, she asked how this could be combined with organ donation for transplantation and SBP. The clinical team had never previously discussed organ donation with her. No protocol existed for this scenario. There was a consensus of hospital administrators and the German Organ Procurement Organization (Deutsche Stiftung Organtransplantation, DSO) that controlled donation after circulatory death (cDCD [10]), the requisite procedure for solid organ transplantation following assisted dying, classified internationally as Maastricht category V (medically assisted circulatory death [10–12]), was not legally established in Germany and thus not feasible.

The patient was informed that donation of vital organs could not be performed due to legal uncertainty. The local ophthalmology department accepted the patient’s offer to recover the corneas. It was therefore suggested to the patient to perform an autopsy for recovery of the brain for SBP per IRB-approved protocol for long-term preservation of the brain, along with the spinal cord for research purposes and the corneas. The patient gave written informed consent to this approach.

The patient died by assisted suicide in the following week at her home. Non-natural death was determined by an independent physician, upon which the police and public prosecutor issued a preliminary release certificate, and the body was transferred to the pathology department and cooled to 0 °C within 6 h of death. The autopsy could be performed the following morning, 20 h after death.

During autopsy, CSF was obtained via lumbar puncture and pH was measured at 6.83, which was previously reported as a positive predictor of ultrastructural quality [13]. Following bilateral enucleation, the spinal cord was extracted and divided into samples for snap-freezing and fixation. The brain was extracted and immersion-fixated for 2 weeks in 4% paraformaldehyde (PFA) at 4 °C [14]. The body was cosmetically restituted and returned to the family for funeral.

**Fig. 2:**
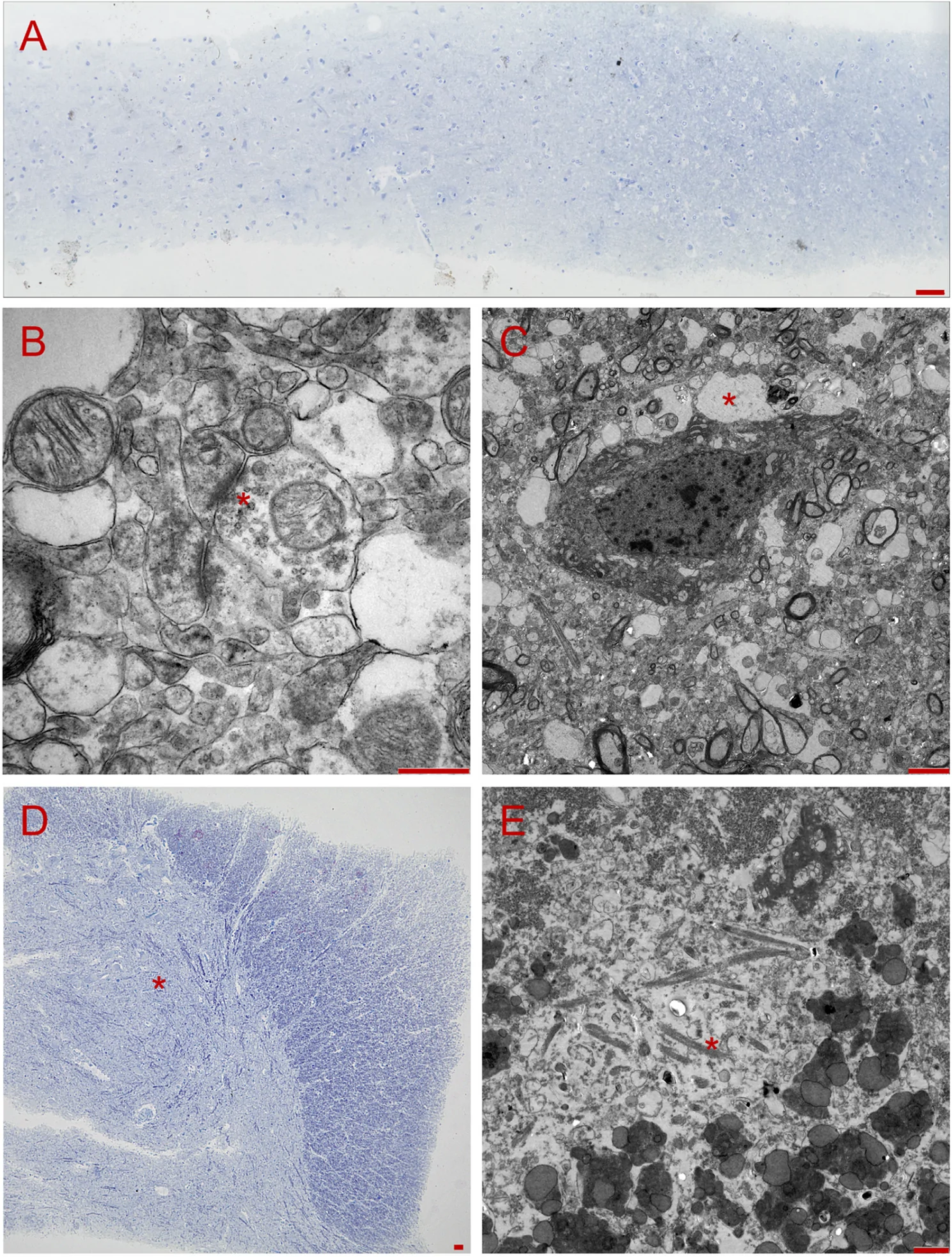
Microscopic analysis of brain specimens. **A** Toluidine blue semithin section of neocortical punch cylinder. Scale bar 100 μm. **B**, **C** Electron microscopy from layer V. **B** Synaptic densities and vesicles (red asterisk) Scale bar 0.5 μm. **C** Dark neuron with marked vacuolization of the neuropil. Scale bar 2.5 μm. **D** Toluidine blue semithin section of the lumbar anterior horn of the spinal cord (red asterisk). Scale bar 100 μm. **E** Electron microscopy showing cytoplasmic linear wisps in spinal anterior horn cell suggestive of TDP-43 aggregates (red asterisk [17]), . Scale bar 1 μm

Minimally-invasive examination of the cerebral nanostructure was performed according to the approved protocol and informed consent: After two weeks of immersion fixation, a minimally-invasive 0.75 mm punch biopsy (Core Sampling Tool, Electron Microscopy Sciences, outer diameter 1.07 mm) was taken from the occipital pole and immersed in the fixative of Ito and Karnofsky [15] for toluidine blue semithin sections and electron microscopy analysis (Fig. 2A). Although synaptic connections and vesicles were still discernible (Fig. 2B), the overall tissue ultrastructure exhibited extensive vacuolization of the neuropil (Fig. 2C). The spinal cord tissue was processed conventionally for histological and ultrastructural analysis (Fig. 2D, E).

The brain was subsequently immersed in vitrification solution and is scheduled for permanent cryogenic storage at the central biobank of the university, at -150 °C to avoid thermomechanical cracking [16].

## Background

The integration of assisted dying into medical practice remains a subject of intense international controversy. The broad spectrum of practices, along with terminological heterogeneity, is also a potential source of confusion. In 2019, the World Medical Association (WMA) specified that “euthanasia is defined as a physician deliberately administering a lethal substance or carrying out an intervention to cause the death of a patient with decision-making capacity at the patient’s own voluntary request. Physician-assisted suicide refers to cases in which, at the voluntary request of a patient with decision-making capacity, a physician deliberately enables a patient to end his or her own life by prescribing or providing medical substances with the intent to bring about death”, and that the WMA is “firmly opposed to euthanasia and physician-assisted suicide” [18]. In 2015, the German Federal Parliament passed criminal legislation with the goal of prohibiting professionalized assisted dying services (§ 217 German Criminal Code). The new law was rendered void by a judgment of the German Federal Constitutional Court in 2020, stating that:

“*It is impermissible to pursue the aim of disapproving*,* placing under a taboo or framing as inferior in any other way the decision of a holder of fundamental rights*,* who acts of their own free will and in personal autonomy*,* to deliberately end their own life with the assistance of others*.” (No. 234, official English translation by the court).

The decision was not limited to terminal illness, as long as the decision is permanent, founded in reality and without external pressure:

“*The right to determine one’s own life*,* which forms part of the innermost domain of an individual’s self-determination*,* is in particular not limited to serious or incurable illness*,* nor does it apply only in certain stages of life or illness*.” (No. 210, official English translation by the court).

This broad permissive judgment raises important policy questions concerning end-of-life practices for Germany and other states with constitutions based on human rights, dignity and self-determination [19]. Discussing these questions in Germany is fraught with historical burden; the term “euthanasia” was abused to describe the systematic killings performed by German healthcare personnel during the National Socialist regime, with very few prominent medical professionals openly objecting [20]. The – often confusing – term *direkte aktive Sterbehilfe*, literally “direct active help in dying” is currently preferred in the German language when referring to voluntary medical euthanasia in human medicine. However, as in other German-speaking countries like Austria and Switzerland, only assisted suicide is legal, while euthanasia has remained punishable as killing on request pursuant to § 216 of the German Criminal Code. Within Europe, Germany currently belongs to intermediate legal systems which permit suicide assistance only, like Switzerland, Austria and Italy, as opposed to active euthanasia in the Benelux countries and Spain at the one end, and strict prohibition at the other end, such as the Claeys-Leonetti framework in France, which only allows deep continuous sedation until death [21–24]. We use the term assisted dying in a broad sense to encompass practices referred to as medical assistance in dying (MAiD), such as physician-assisted suicide (PAS) as defined by the WMA, physician-assisted dying (PAD), or euthanasia as defined by the WMA in regional contexts, denoting the voluntary and supervised ending of life at the explicit request of a competent person [25].

Here, we report our experience with assisted suicide with a particular focus on neurodegenerative disorders. Confronted with terminal patients’ requests for assisted dying, we aim to highlight potential policy considerations in connection with organ and tissue donation for transplantation, research, and long-term preservation practices like Structural Brain Preservation and cryonics, which we will outline next.

## End-of-life considerations in the context of neurodegeneration

### Prognosis and assisted dying

Neurodegeneration is the second most frequent reason, after cancer, for assisted dying in the Netherlands, where assisted dying has been legally regulated and systematically reported for over two decades [26]. A similar pattern is emerging in Germany [27]. In neurodegenerative conditions like amyotrophic lateral sclerosis (ALS) and multiple system atrophy (MSA), the relative proportion of patients seeking assisted dying is particularly high with 31% of ALS patients requesting assisted dying in a prospective Dutch cohort [28], and decision-making capacities commonly preserved in advanced stages of disease [29]. Unlike cancer, most neurodegenerative diseases are lethal and incurable from the time of diagnosis, prompting the initiation of advance care planning. In the Netherlands, 25% of deaths from ALS between 2012 and 2020 had involved assisted dying [26]. This choice did not lead to a significant reduction of overall survival time compared to patients who did not undergo assisted dying, indicating that assisted dying was sought at advanced disease stages, when alternative end-of-life practices are also frequently applied [26].

### Organ donation for transplantation

Unlike most types of cancer, many neurodegenerative patients are medically eligible for organ donation for transplantation [6, 30]. This requires assisted dying, however, since death, e.g., from ALS, otherwise occurs outside the circumstances required for vital organ donation, with sudden and unpredictable terminal decline involving organ failure [31]. Organ donation in assisted dying results in a range of benefits for transplant recipients:

- Scarcity: The number of available organs is insufficient to meet the needs of patients with end-stage organ failure. Among listed transplant candidates, a substantial proportion die while awaiting transplantation [32]. This shortage is further exacerbated by increasing demand and declining supply of donor organs, partly attributable to a reduced incidence of traumatic brain injury [33]. Multiple approaches to alleviate the organ scarcity have been proposed, including opt-out rules, xenotransplantation, bodyoids, and organ donation euthanasia in intensive care [34–36]. Donation in the context of assisted dying has the potential to increase the number of available donor organs [37, 38]. Annual ALS and assisted dying cases both exceeded the number of organ donors in Germany in 2024 [39].
- Effectiveness: Similar to donation after brain death, up to nine transplants can be gathered from one organ donor (1 heart, 2 lungs, 2 kidneys, 2 split-livers, 1 pancreas, and 1 intestine).
- Safety and Quality: Organ engraftment following donation after assisted dying exhibited a favorable safety profile [40–44]. This is possible due to advance planning and short ischemia times, and commonly good extracerebral health status in neurodegeneration [40].

For the donor, donation is not medically beneficial. Its value lies in the realization of an autonomous altruistic choice. Many general objections to assisted dying arise from its potential harms to the patient and their family. In the specific context of organ donation, these potential harms include:

- Hastened death: Patients with neurodegeneration might hasten assisted dying, e.g., to benefit recipients or due to collective pressure, and forgo remaining quality of life.
- Abuse and diagnostic uncertainty: Clinicians might render the diagnosis of, e.g., a neurodegenerative disease prematurely, due to diagnostic error or the aim of increasing organ availability. This has already prompted legal action, e.g., in the case of Ruben Navarro [34].
- Medicalized death: Assisted dying in connection to organ donation in a hospital setting, or an operating theatre, might be perceived as the complete medicalization of the dying process, which is opposed by positions emphasizing naturalness [45] and could negatively affect the dying experience of the patient and grieving process of the family. Organ donation after euthanasia starting at home has been performed in an attempt to address this issue [46].

A general concern in transplantation includes the Kantian rule not to use people as a mere means to an end. However, safeguarding individual autonomy means that the donor is no longer used merely as a means, but also as a chooser of ends in herself.

Policy to avoid the described harms might include, e.g., independent review and exclusion of monetary interests or psychological pressure by separating the assisted dying and organ donation procedures and forestalling that donating organs is the primary goal of hastening death [4]. For example, while patients with progressive terminal illnesses may inevitably contemplate both options concurrently during advance care planning, policy can require that the formal approval for assisted dying is finalized before clinical evaluation or consenting for organ donation begins. Furthermore, this separation can be reinforced by ensuring that the healthcare professionals evaluating the request for assisted dying are independent from the transplant procurement team.

### Research

The etiology of most neurodegenerative diseases is poorly understood and difficult to model experimentally, compared to, e.g., cancer cell lines in vitro. Brain biopsies are rarely performed due to their invasiveness and lack of immediate therapeutic implications for the patient, attaching great importance to the practice of obtaining biomaterials for research post-mortem. Short ischemia tolerance of brain tissue, along with the increased sensitivity of modern analysis techniques like RNA sequencing, make the immediate donation of brain tissue in the context of assisted dying particularly valuable for advancing the understanding of neurodegenerative conditions and development of potential cures. Similar counterarguments and concerns as in organ donation apply to research donation.

### Structural brain preservation and cryonics

Preservation practices like Structural Brain Preservation (SBP) and cryonics are motivated by the hope that the brain will become readable by structural analysis in the future, and its function re-instantiated via advanced technologies, e.g., molecular nanotechnology [47] or whole brain emulation [48]. Preservation techniques include tissue stabilization via vitrification in the state of a cryogenic glass below − 130 °C [49–51] and via chemical crosslinking to a gel via aldehyde fixation [52]. Cryogenic and chemical methods may also be combined [53]. The common goal of these techniques is to preserve the biomolecule-annotated nanoscopic connectome, in the belief that it may contain necessary and sufficient information about determinants of individual personal identity [47]. SBP cannot currently be evaluated by standard clinical efficacy endpoints and therefore requires a translational evidentiary approach based on surrogate preservation endpoints. For justifications of performing SBP/cryonics despite their lack of clinical evidence at the end of life, see Shaw [54], Moen [55] and Thau [56].

SBP/cryonics exist along a gradient of scope regarding the biological systems retained, from neuropreservation (brain or head-only), neuro-motor preservation (extension to the spinal cord, peripheral nervous system and the musculoskeletal system), to whole-body preservation. Whole-body preservation is inherently mutually exclusive with the donation of vital organs, whereas neuropreservation and neuro-motor preservation remain compatible (Table 1). Optimizing the nanoscopic quality of the brain structure is a key consideration when performing SBP/cryonics, which has similar constraints on ischemia time as those imposed by transplantation and research aims. The preservation quality will therefore be increased in assisted dying situations compared to most other causes of death.

**Table 1:** Procedural compatibility of end-of-life donation and preservation options

Option	Organ donation after brain death	Organ donation after circulatory death	Whole-brain donation	Brain biopsies	Peripheral biopsies	Whole-brain preservation	Neuro-motor preservation	Whole-body preservation
Organ donation after brain death	-	incompatible	compromised	compromised	restricted	compromised	compromised	incompatible
Organ donation after circulatory death	-	-	compatible	compatible	restricted	compatible	compatible	incompatible
Whole-brain donation	-	-	-	compatible	compatible	incompatible	incompatible	incompatible
Brain biopsies	-	-	-	-	compatible	restricted	restricted	restricted
Peripheral biopsies	-	-	-	-	-	compatible	restricted	restricted
Whole-brain preservation	-	-	-	-	-	-	compatible	compatible
Neuro-motor preservation	-	-	-	-	-	-	-	compatible
Whole-body preservation	-	-	-	-	-	-	-	-

Compatibility matrix for organ donation, tissue sampling for research, and preservation options. Entries indicate whether two options are technically compatible, technically incompatible, feasible only with compromised tissue quality, or restricted by the scope and anatomical location of tissue sampling

The outcome of present SBP/cryonics methods is death according to medico-legal standards, but hope for avoidance of death according to an “information-theoretical criterion” [57]. In 2013, legal scholar and medical ethicist Jochen Taupitz, then a member of the German Ethics Council and of the Central Ethics Commission of the German Medical Association, concluded that SBP/cryonics is, in principle, compatible with German law, when weighing post-mortem human dignity, the human dignity of the living, the autonomous wish for cryopreservation, and the implications of a prohibition of SBP/cryonics [58]. 22% of Germans reported that they would consider cryonics procedures for themselves in a representative survey from 2014 [59], and its utilization in Germany has recently surpassed that of rare clinical interventions, such as limb transplantation [60]. In a 2025 survey of U.S. physicians on SBP/cryonics as an end-of-life option, a median 25.5% probability was assigned that preservation performed under ideal conditions could retain neural information sufficient for potential future recovery; 27.9% of physicians rated the concept as somewhat/very plausible and 47.0% as somewhat/very implausible, see Zeleznikow-Johnston et al. [61].

### SBP/cryonics as a reinterpretation of the assisted dying debate

The permissibility of assisted dying has remained highly contested since the inception of human medicine as an act of ending suffering by ending the existence of the sufferer [25]. Prominent objections are grounded in moral frameworks such as the Catholic doctrine of double effect, which rejects actions intended to cause death, and in deontological interpretations of the Hippocratic tradition that prohibit physicians from intentionally killing patients. Shaw [54], Moen [55], Minerva and Sandberg [62], Andrade and Redondo [63] and Buben [64] have argued that SBP/cryonics reinterprets the dilemma between suffering and killing [65]. According to this line of reasoning, because SBP/cryonics are acts intended to preserve, not terminate the existence of patients, they fall outside several of the core objections raised against assisted dying, while still alleviating suffering at the time of intervention.

Against this, it is conceptually helpful to distinguish between assisted dying with the primary goal to end suffering, to end a completed life or to avoid the alteration of personal identity due to progressive brain damage, e.g., by dementia, and assisted dying with the primary goal to optimize preservation quality for SBP/cryonics (“dying to live”) [62, 63], discussed in the academic literature under the terms “cryothanasia” and “cryocide” in relation to the cases of Thomas Donaldson [66], Kim Suozzi [62] and Norman Hardy [67].

### Legal and procedural barriers to tissue quality

The integration of organ and tissue donation and SBP/cryonics into assisted dying faces significant legal and procedural barriers. In Germany, any non-natural death, which includes assisted dying, must be reported to the police and public prosecutor. The authorities then decide whether to mandate a forensic autopsy or to issue a body release certificate (*Freigabebescheinigung)* allowing burial or other disposition. An autopsy, if required, would delay organ/tissue recovery and physically disrupt the CNS, defeating the transplantation, research, and SBP/cryonics aims [68]. German legislation regarding organ transplantation currently requires death and irreversible loss of brain function (ILBF). The German Medical Association has specified the protocols for the determination of ILBF, which is regarded sufficient for the determination of death of the human being. According to this protocol, donation after circulatory death (DCD) is currently only feasible after 3 h or more of circulatory arrest which exceeds ischemic tolerance of most organs by far and practically excludes DCD (except, for e.g., the cornea) [69].

## Discussion

### Case discussion

Tissues from vital organs except the central nervous system and the cornea were not utilized in the present case. This constitutes a partial realization of the wishes of the patient. While the request for assisted dying was met, the requests for organ donation for transplantation and brain preservation were addressed incompletely. From the patient’s point of view, notwithstanding the outlined legal barriers, realizing her wishes to a complete extent would have increased the self-determination and potentially benefited other patients, directly via transplantation, or indirectly via availability of research materials obtained during cDCD.

Two similar cases of assisted dying in ALS and in MSA have occurred in patients under our care. Brain donation was only possible more than 24 h post-mortem in these cases, due to delays at the stage of the mortician and public prosecutor, respectively. Their brain tissue showed marked autolysis on gross examination. Accordingly, the requests of these patients were only met in form but failed in substance. Although some brain structure can be retained even after prolonged cold storage, short post-mortem times are generally required to guarantee good preservation of tissue [70].

### General discussion

The 2020 judgment of the German Federal Constitutional Court has affirmed far-reaching rights of self-determination, including access to assisted dying for competent adults who persistently wish to end their lives. It reinstated the right to assisted suicide that had existed in Germany since 1871 up to legislation in 2015 and endowed it with an added measure of publicity (Federal Constitutional Court judgment, No. 17). At the same time, neither German guidelines nor statutory regulation currently specify a pathway when such patients also wish to donate organs, contribute tissue for research, or undergo SBP/cryonics. Our case suggests that this uncertainty can result in only a partial realization of patient wishes despite advance planning.

This gap gives rise to several policy questions: (i) How should assisted dying itself be regulated in a setting where it is constitutionally protected but remains contested? (ii) Is it permissible, and under what safeguards, to combine assisted dying with organ transplantation, tissue banking, or SBP/cryonics?

Professional German guidelines on suicide prevention and the handling of requests for assisted dying focus on palliative, psychiatric, and communicative obligations [71, 72], as do recommendations by the German Ethics Council [73]. They do not explicitly address questions (i)-(ii).

The 2020 judgment of the German Federal Constitutional Court comes at a time when SBP/cryonics have been performed more frequently since 2019, with multiple procedures reported by preservation providers like the Alcor Life Extension Foundation, The Cryonics Institute [74], Tomorrow Biostasis GmbH [75] and our center (www.uker.de/mn-hirnarchiv, IRB-approval No. 23-416-B, 02/29/2024). This situation has given rise to the question (iii) whether the judgment also pertains to assisted dying sought with the primary or partial intent to optimize preservation quality in SBP/cryonics. In principle, the judgment grants applicability to a broad spectrum of intentions:

“*What is decisive is the will of the holder of fundamental rights*,* which eludes any appraisal on the basis of general values*,* religious precepts*,* societal norms for dealing with life and death*,* or considerations of objective rationality*” (No. 210, official English translation by the court).

However, while current SBP/cryonics techniques inherently result in death under codified medical and legal definitions, they may avert death under the “information-theoretic” criterion, which requires irreversible loss of brain structure. Accordingly, while the 2020 judgment protects the freedom to end one’s life with subsequent irreversible destruction of brain structure, it may not extend to ending one’s life in a manner that preserves that structure.

Arguably, choosing SBP/cryonics may be understood to constitute a ‘minus’ in comparison to other forms of assisted dying: If ending one’s life followed by irreversible destruction of brain structure is covered by the exercise of personal autonomy, why would the decision to end one’s life followed by preservation of that structure not be. If the comprehension necessary to express informed consent in regard to ending one’s life followed by irreversible destruction of brain structure is deemed possible, the same must apply to the comprehension of the concept of ending one’s life followed by preservation of that structure, and thus of SBP/cryonics. After all, while it is just as inconceivable, it may present less of an interference with existence as such, see [76] pp. 398 ff. In the hypothetical case that SBP/cryonics would not be legally classified as an act of ending one’s existence, even stronger constitutional protection of self-determination and life could be presumed [58].

While the domestic and international academic discussion on medicalization vs. de-medicalization of assisted dying is ongoing [77], the challenges arising at the intersection with organ transplantation, tissue banking and SBP/cryonics require specialized medical expertise for their safe and reliable execution and remain closely connected to core functions of medical practice.

Professional standards and procedural consistency in addressing these challenges in the context of assisted dying could be enhanced through structured planning and standardization, rather than reliance on ad hoc, case-by-case approaches. Considerable effort, cost and delays are caused by the coordination of fragmented stakeholders in the ambiguous policy landscape, including the caregiving, assessment of the patient’s decision, assistance in dying, death certification, police, public prosecutor, coroner, mortician, transplant surgeon, researchers and SBP/cryonics practitioners. In light of the present case-related experiences, we provide a hypothetical proposal for legislative reforms addressing questions (i)-(iii) and to streamline the activities of these traditionally fragmented stakeholders to realize the wishes of patients undergoing assisted dying (Donation-Preservation Pathway, DPP, Table 2).

**Table 2:** Proposal for a Donation-Preservation Pathway (DPP) in assisted dying

A dedicated care unit provides assistance for patients with an independently evaluated and approved choice to undergo assisted dying under the applicable legal framework. Informed consent detailing the purposes and compatibilities of all requested donation and preservation procedures is obtained after a reflection period. The unit provides the setting for a self-determined death under legal oversight and forensic documentation. Subsequently, the patient is transferred to an operating theatre to perform the procedures requested by the patient. In the prototypical case, this may include 1) vascular perfusion with cold organ preservation solution, 2) explantation of vital organs for pre-scheduled transplantation into recipients with organ failure, ideally at the same transplant center (i.e., Maastricht V DCD), 3) research biopsies for tissue banking, 4) SBP/cryonics via vascular perfusion with fixatives/cryoprotectants and transfer to a storage facility, e.g., a local biobank.	

The full DPP does not comply with current legislation in Germany. Legislative and organizational steps could be undertaken to ensure professional standards when realizing requests by patients choosing assisted dying. This might entail inclusion of assisted dying and DCD in transplantation law, as is currently discussed in the scientific community [78–80] but has not yet found its way into the parliamentary debate [81]. A key consideration when undertaking such steps will be community acceptance, and the maintenance of public trust in traditional types of organ donation, as outlined by Bollen et al. [82].

Some variations of DCD and the DPP may violate the dead-donor rule (DDR) for organ transplantation, although this rule has been criticized [83, 84] and does not apply to living donation [34, 85]. We identify variants (i)-(iv) of the DPP with respect to the DDR below:

(i) A pathway may fulfill the DDR with hour-long post-mortem waiting periods that ascertain irreversible loss of brain function but severely compromise organ and tissue quality, ruling out DCD. This is the present legal situation in Germany [80, 86].
(ii) One may realize controlled DCD after assisted dying (Maastricht V) while respecting the DDR with a short no-touch period that rules out spontaneous recovery. This is the present situation in most jurisdictions enabling DCD and would be sufficient to enable the full scope of the DPP.
(iii) A DDR-reinterpreting version of (ii) would omit the no-touch period after assisted dying in order to reduce organ damage. In support of this, Patuzzo Manzati et al. have pointed out that in assisted dying/Maastricht V situations, the donor may be understood as irreversibly dying, akin to entering a metaphorical “tunnel without return” toward death at the timepoint of administration of life-terminating drugs [87].
(iv) More fundamental DDR-revising versions omit the intermediate step of assisted dying as a separate, life-ending act altogether and instead allow organ retrieval or preservation itself to be life-ending. This no longer constitutes conventional DCD and has been discussed as organ donation euthanasia [34] and euthanasia through living organ donation for transplantation [82], as well as cryothanasia [62] and cryocide [88] for SBP/cryonics, respectively. In version (iv), only the self-administered loss of consciousness, but no longer the self-administration of life-terminating drugs would be practically realizable.

SBP/cryonics raise legal and ethical questions with respect to the status, storage obligations and long-term stewardship of the preserved specimens. The importance of safe storage might then be comparable to, e.g., cryopreserved specimens of autologous stem cells during high-dose chemotherapy, which are vital for marrow rescue. A brief analysis of the DPP according to established models in professional medical ethics is in order [89].

- Autonomy: The DPP could increase the options available to patients choosing assisted dying, transplantation, research and SBP/cryonics, thereby increasing their autonomy. Opponents might find that the autonomy granted by DPP goes too far, e.g., in light of modern knowledge about the cognitive presuppositions of advance directives, postmortem instructions and legacies [90–92].
- Justice: Organ and research donation promote equity by contributing to public health, and a DPP could alleviate current wealth-dependent accessibility gaps in SBP/cryonics but would rely on public resources.
- Beneficence: On an aggregative view, the DPP could increase the availability of transplantable organs and human biomaterials for research. This could benefit organ recipients and future patients via research. Benefits to the patient are achieved if suffering is relieved and SBP/cryonics results in the desired outcome. Opponents might object that the risk of disproportionately curtailing the life of patients is too high.
- Non-maleficence: The opportunity to donate organs might induce vulnerable patients to end their lives prematurely and to forgo chances to live a period of life with good or at least satisfactory quality of life. Erosion of trust in the medical profession might ensue in the public due to the perceived instrumentalization of assisted dying by making use of the organs of the deceased for others or for research purposes. Long-term uncertainties inherent in SBP/cryonics might entail unknown harms [56, 93, 94].

An important consideration for non-maleficence is systemic anticoagulation, typically heparin [4]. From a transplantation perspective, pre-mortem heparinization can improve graft quality by reducing thrombotic microangiopathy. Since it does not relieve symptoms and increases the risk of hemorrhage, it can be viewed as a purely recipient-oriented intervention. From the perspective of SBP/cryonics, patency of the microvasculature is a prerequisite for perfusion with cryoprotectants or fixatives, thus turning heparin into a donor-oriented intervention. Zeleznikow-Johnston et al. found that 70.7% of U.S. physicians supported prescribing anticoagulants for this indication, while 11.7% opposed it [61].

The DPP could in theory be realized by a sanctioned integrated provider, e.g., one national transplant center with a cooperating palliative care unit and public prosecutor. Offers for palliation could cushion distress inherent in the DPP for patients and families. Palliative medicine has been instrumental in challenging a purely survival-oriented medical paradigm, prioritizing the alleviation of suffering and the acceptance of dying as an integral part of life [45]. Valid concerns persist that institutionalizing assisted dying alongside standard care risks blurring the normative distinction between accompanying the dying and actively hastening death [95, 96]. A DPP should therefore be designed not to co-opt palliative care, but potentially allow for a synthesis where participation is voluntary.

Concerning the professionals involved in a DPP, any concrete implementation would have to take seriously the emotional impact, similarly to the fields of palliative care and emergency intensive care medicine. Empirical work on assisted dying suggests that participation can be experienced as both meaningful and burdensome, with frequent reports of moral distress, ambivalence and risk of burnout [97, 98]. Integrating assisted dying, organ donation and SBP/cryonics into a single pathway would therefore require consideration of the broader debate on conscientious objection in health care [99–101]. Professional attitudes toward assisted dying, organ donation, and SBP/cryonics are heterogeneous. Some professionals may support assisted dying and organ donation separately but object to combining them, because organ donation could instrumentalize the dying person or unduly normalize assisted dying. Others may accept organ donation or SBP/cryonics, but will only regard assisted dying as acceptable when it is linked to such procedures, because they see the gains for organ recipients or the hope of future survival as outweighing the costs. Conversely, still others may worry that performing organ donation or SBP/cryonics in connection with assisted dying introduces problematic motives. Statutory regulation for non-participating staff may be gleaned from other jurisdictions like Queensland [102], as well as expertise from existing training programs and model practice standards for participating staff [103].

Concerning the patients involved in a DPP, risks of coercion, for example through social expectations “*to give back*” or economic hardship [104] could be amplified by the prospect of combination with organ donation or SBP/cryonics. Independent review could help in ensuring decisions for assisted dying are only made after the patient has had access to high-quality palliative care and psychosocial support to enhance self-determination and avoid premature decisions. The tension between maximizing remaining life vs. donation/preservation quality may need safeguards to exclude misconceptions and unstable motivations, similar to the psychological evaluation of living organ donors [105–107]. Patients should be able to withdraw from assisted dying altogether or from individual elements of the DPP at any point.

Policy design must also address who bears the costs of a DPP. In some jurisdictions assisted dying procedures are funded through existing public schemes, with cost analyses suggesting that the direct costs may be offset by reduced expenditures on end-of-life care [108, 109]. Of note, referring to these analyses is not intended as a justification for assisted dying, but as a contextual consideration for policy design. One plausible approach would be for the DPP to align with existing funding models for end-of-life and transplant services, while requiring separate financing for SBP/cryonics components that exceed standard biobanking. Financing arrangements and safeguards for the professional and responsible administration of SBP/cryonics constitute a distinct policy field that extends beyond assisted dying and requires dedicated legal oversight [110].

### Limitations

The comparison between assisted dying, organ and research donation and SBP/cryonics should not be understood as a claim of moral equivalence. Organ and research donation are primarily other-regarding and carry the risk of external coercion, while assisted dying and SBP/cryonics are primarily self-regarding and carry the risk of internal miscalculation, although morally hybrid scenarios have been constructed [93, 111]. Further important viewpoints, beyond the autonomy-focused analysis performed herein, like the ethics of care [112], social justice [113], planetary health [114], and bodily integrity [115] are warranted for a more substantive philosophical evaluation.

In countries such as Germany, the handling of assisted dying, transplantation, research, and SBP/cryonics is the result of evolving patient preference, public opinion and legislation. Reasonable individuals can be expected to hold differing views about each of these practices and their combinations [116]. We suggest that effort is best directed at establishing pathways that prioritize legal certainty and patient safety by maintaining (i) the legal scope of self-determination, (ii) protection from coercion or neglect, and (iii) equitable access and professional standards in the administration of medical procedures.

## Conclusion

Despite constitutional protection of autonomous assisted dying in Germany and other jurisdictions, fragmented stakeholders and ambiguous legislation lead to delays that complicate transplantation, research, and cryonics or Structural Brain Preservation. A clearly regulated donation and preservation pathway could provide a procedural framework to reduce the risk of misuse and implicit pressure while at the same time increasing the scope of autonomous choices and benefits for research and transplantation.

## Data Availability

All data generated during this study are included in this published article.

## Footnotes

1 Original German wording used during counseling (see www.uker.de/mn-hirnarchiv): „*Durch neue technische Möglichkeiten könnte in Zukunft die Kartierung der Hirnstruktur möglich werden. Die Konsequenzen hieraus sind gegenwärtig nicht absehbar. Es wird spekuliert*,* dass nach Hirnarchivierung in ferner Zukunft das Auslesen der zu Lebzeiten gesammelten Erinnerungen oder eine Wiedererlangung der Hirnfunktion möglich werden könnte.“*.

## Abbreviations

ALS: Amyotrophic lateral sclerosis
ALS-FRS-R: Amyotrophic Lateral Sclerosis Functional Rating Scale–Revised
cDCD: Controlled donation after circulatory death
CNS: Central nervous system
CSF: Cerebrospinal fluid
DCD: Donation after circulatory death
DPP: Donation-Preservation Pathway
DSO: Deutsche Stiftung Organtransplantation
ILBF: Irreversible loss of brain function
IRB: Institutional Review Board
MAiD: Medical assistance in dying
MSA: Multiple system atrophy
PAD: Physician-assisted dying
PAS: Physician-assisted suicide
PEG: Percutaneous endoscopic gastrostomy
PFA: Paraformaldehyde
SBP: Structural Brain Preservation
TDP-43: TAR DNA-binding protein 43
VSED: Voluntary stopping of eating and drinking
WMA: World Medical Association
